# Supplementary figures and images for: mTOR signalling controls the formation of smooth muscle cell-derived luminal myofibroblasts during vasculitis
Source: EMBO Rep. 2024 Sep 13;25(10):4570–93. doi: 10.1038/s44319-024-00251-1 (PMC11467406; doi:10.1038/s44319-024-00251-1)

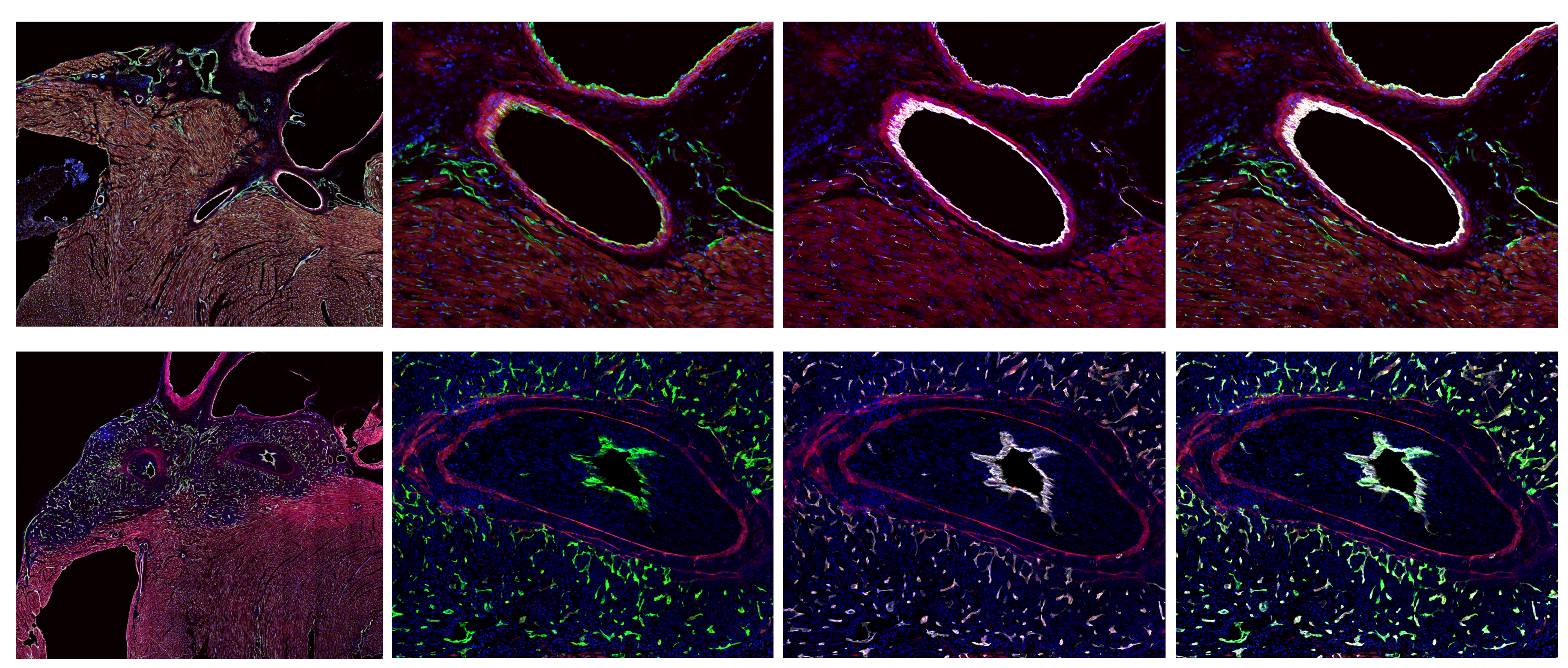

Supplement: Supplementary file 2 — Source data Fig. 1 [file 44319_2024_251_MOESM2_ESM.zip › Fig 1/Fig 1H/1H.tiff]

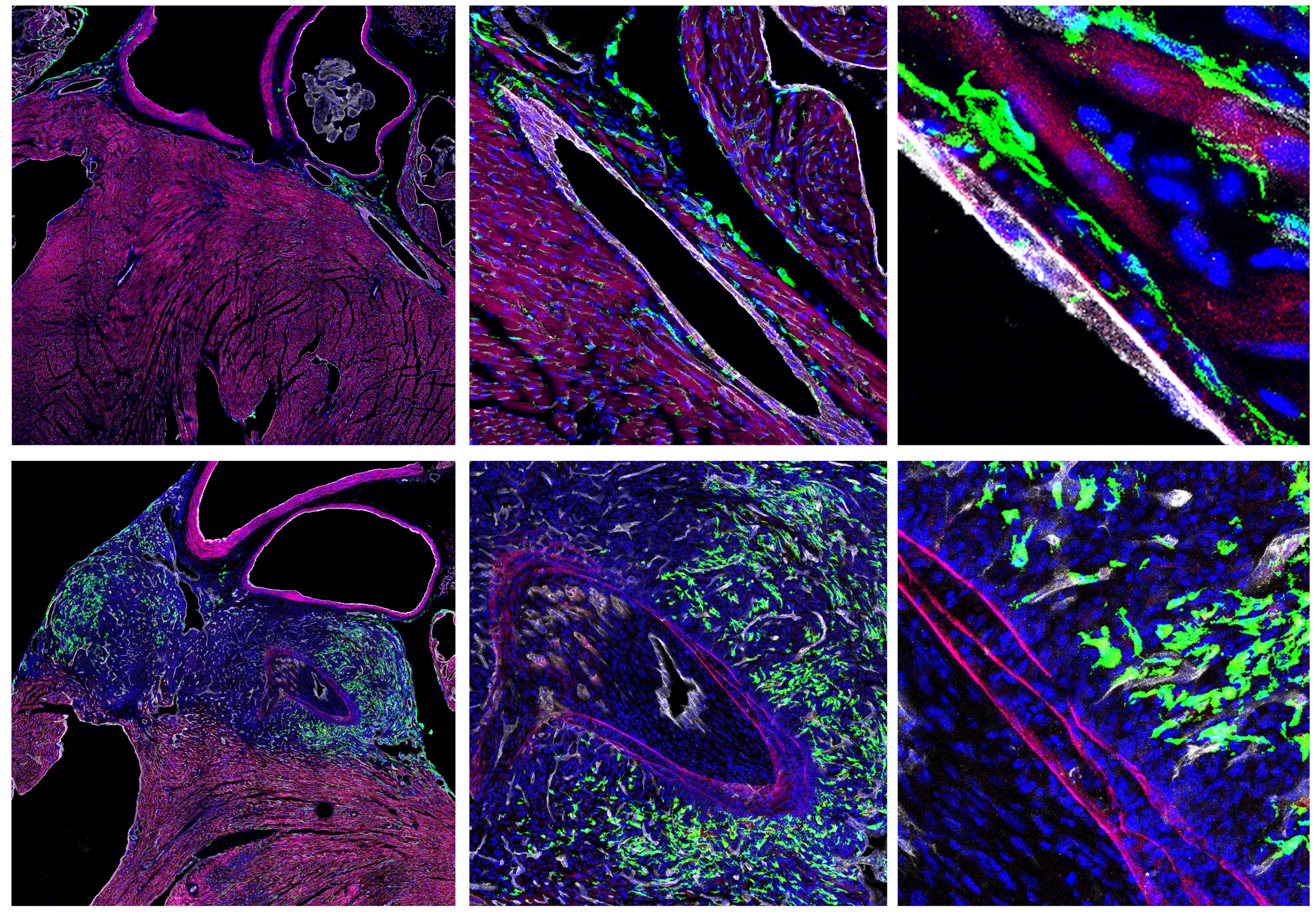

Supplement: Supplementary file 2 — Source data Fig. 1 [file 44319_2024_251_MOESM2_ESM.zip › Fig 1/Fig 1E/1E.tiff]

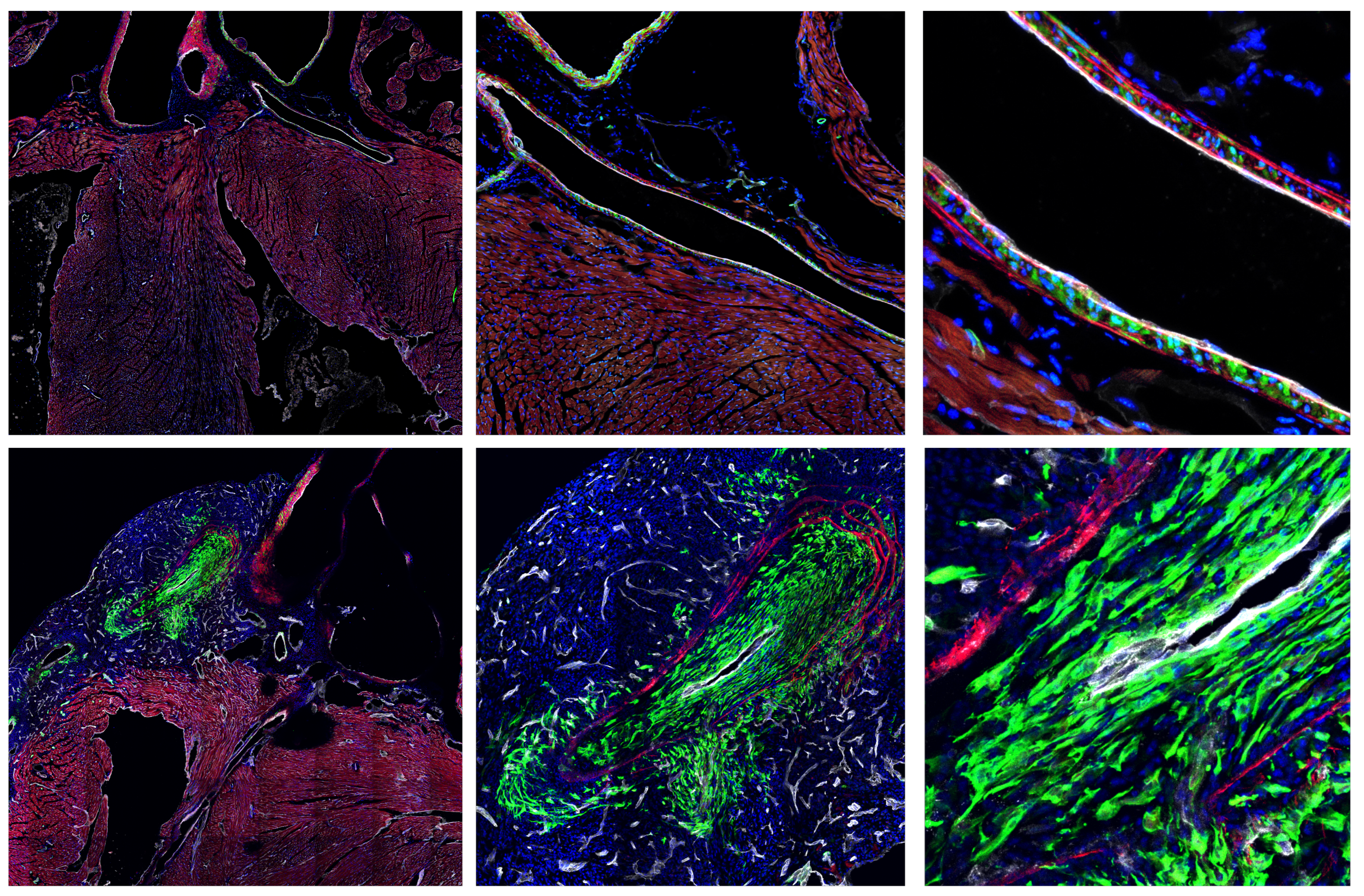

Supplement: Supplementary file 3 — Source data Fig. 2 [file 44319_2024_251_MOESM3_ESM.zip › Fig 2 Source data/Fig 2F/2F.tiff]

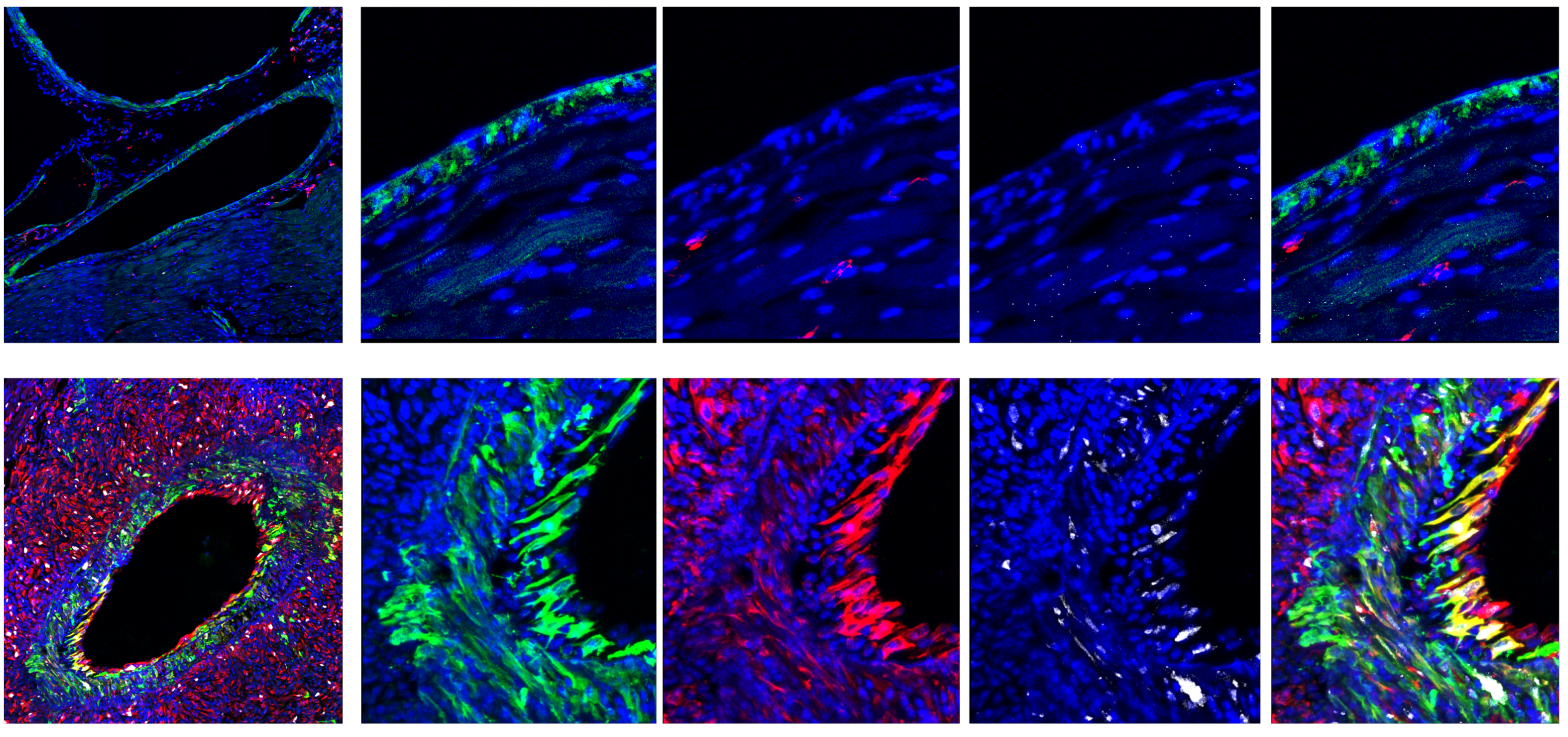

Supplement: Supplementary file 4 — Source data Fig. 3 [file 44319_2024_251_MOESM4_ESM.zip › Fig 3 Source data/Fig 3B/3B.tiff]

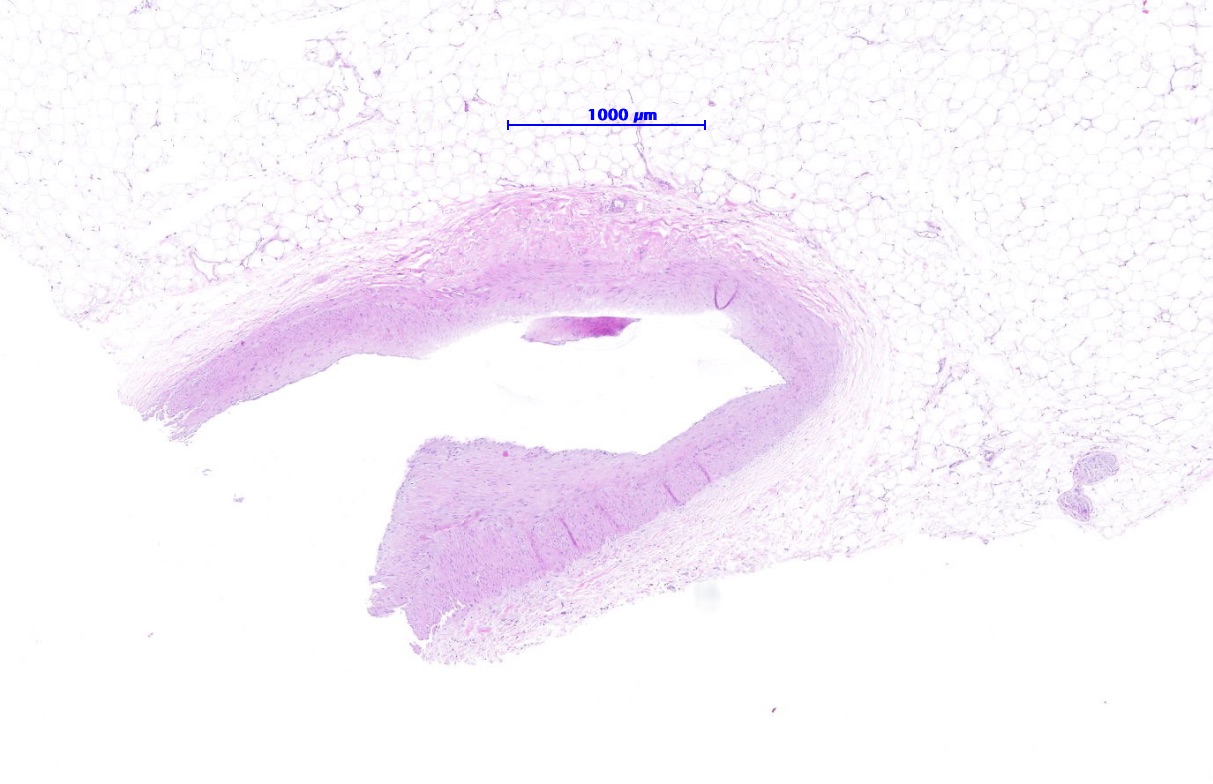

Supplement: Supplementary file 5 — Source data Fig. 4 [file 44319_2024_251_MOESM5_ESM.zip › Fig 4A/control 2.jpg]

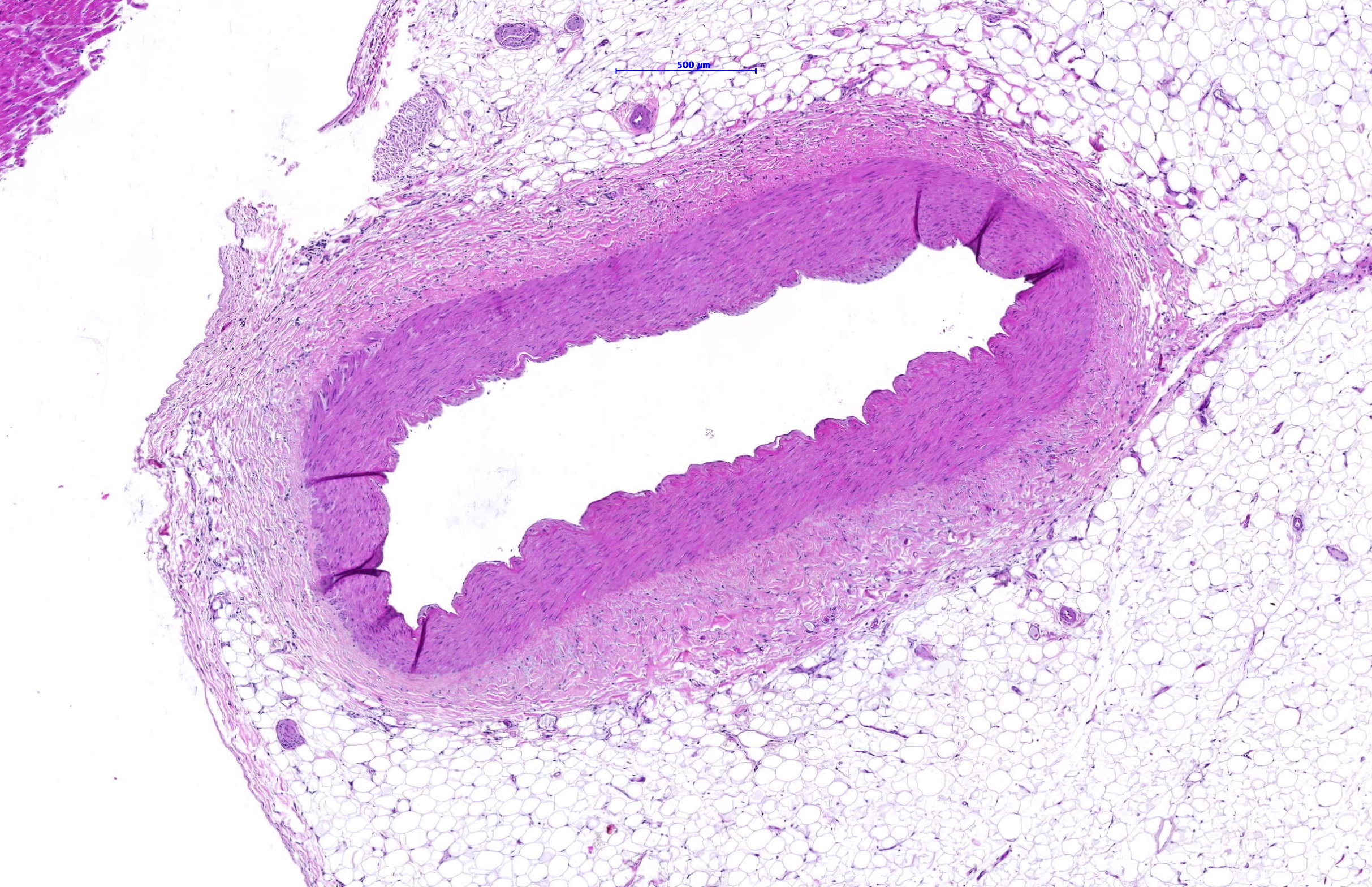

Supplement: Supplementary file 5 — Source data Fig. 4 [file 44319_2024_251_MOESM5_ESM.zip › Fig 4A/control 1.jpg]

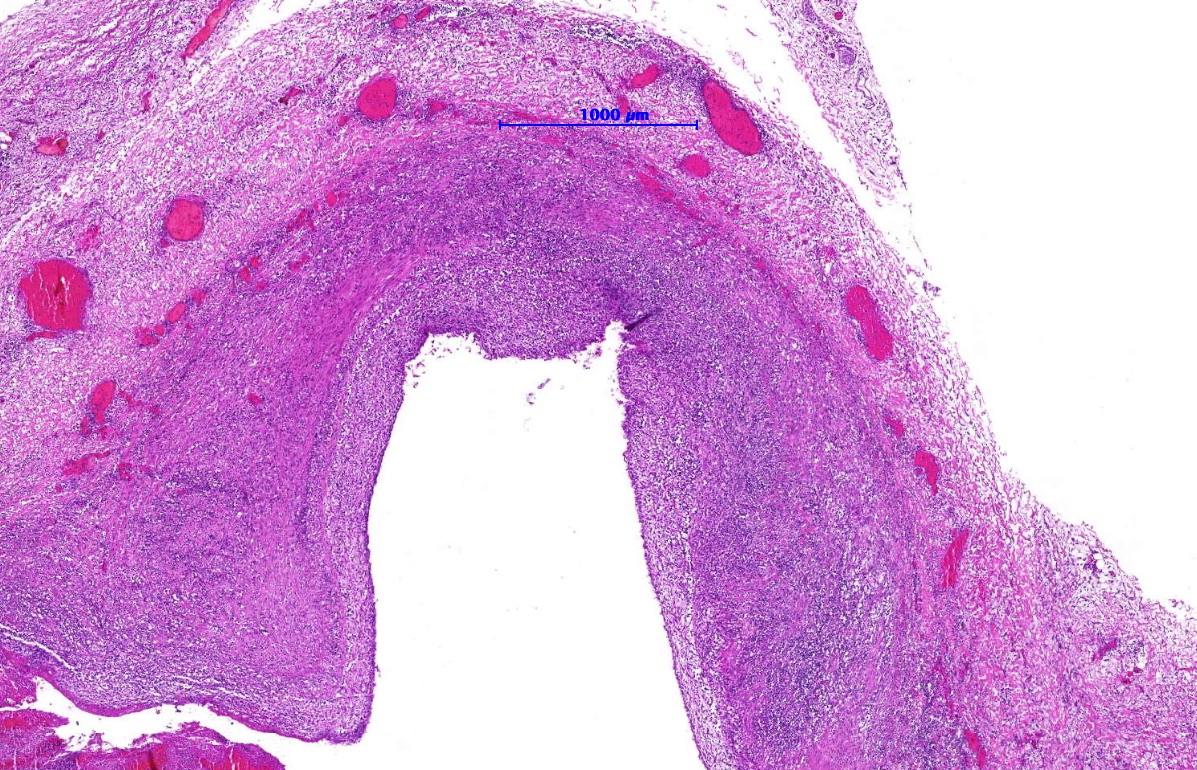

Supplement: Supplementary file 5 — Source data Fig. 4 [file 44319_2024_251_MOESM5_ESM.zip › Fig 4A/KD 1.jpg]

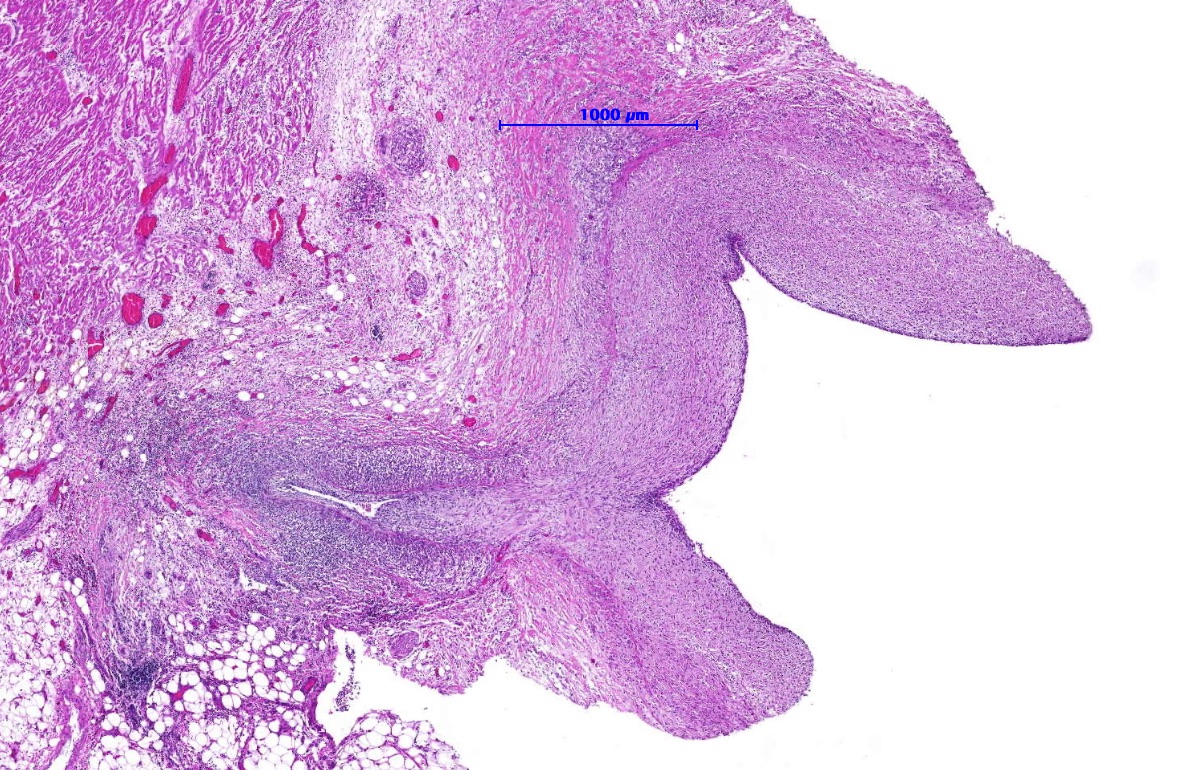

Supplement: Supplementary file 5 — Source data Fig. 4 [file 44319_2024_251_MOESM5_ESM.zip › Fig 4A/KD 2.jpg]

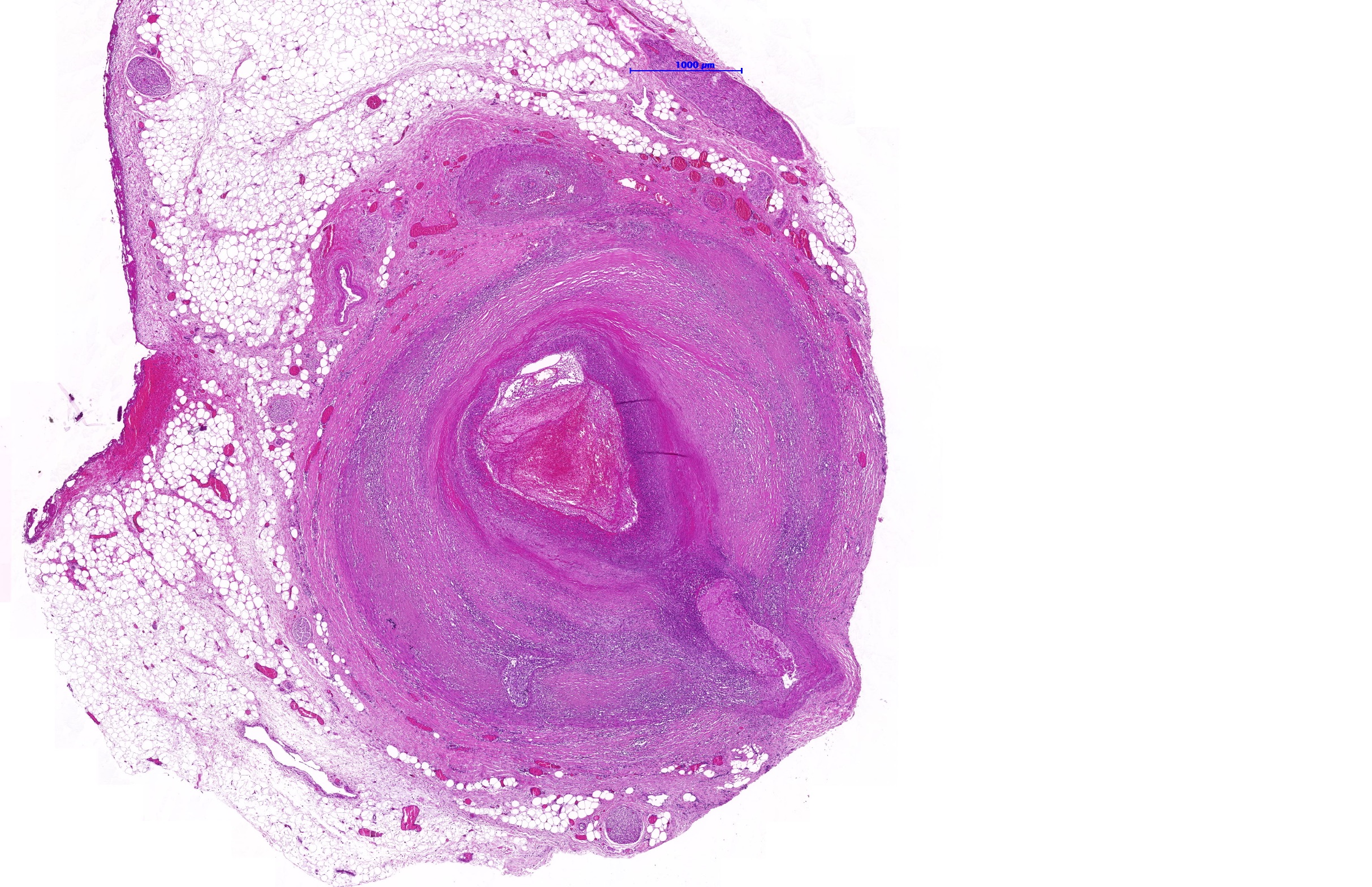

Supplement: Supplementary file 6 — Source data Fig. 5 [file 44319_2024_251_MOESM6_ESM.zip › Fig 5/Fig 5A/TAK 2009 copy.jpg]

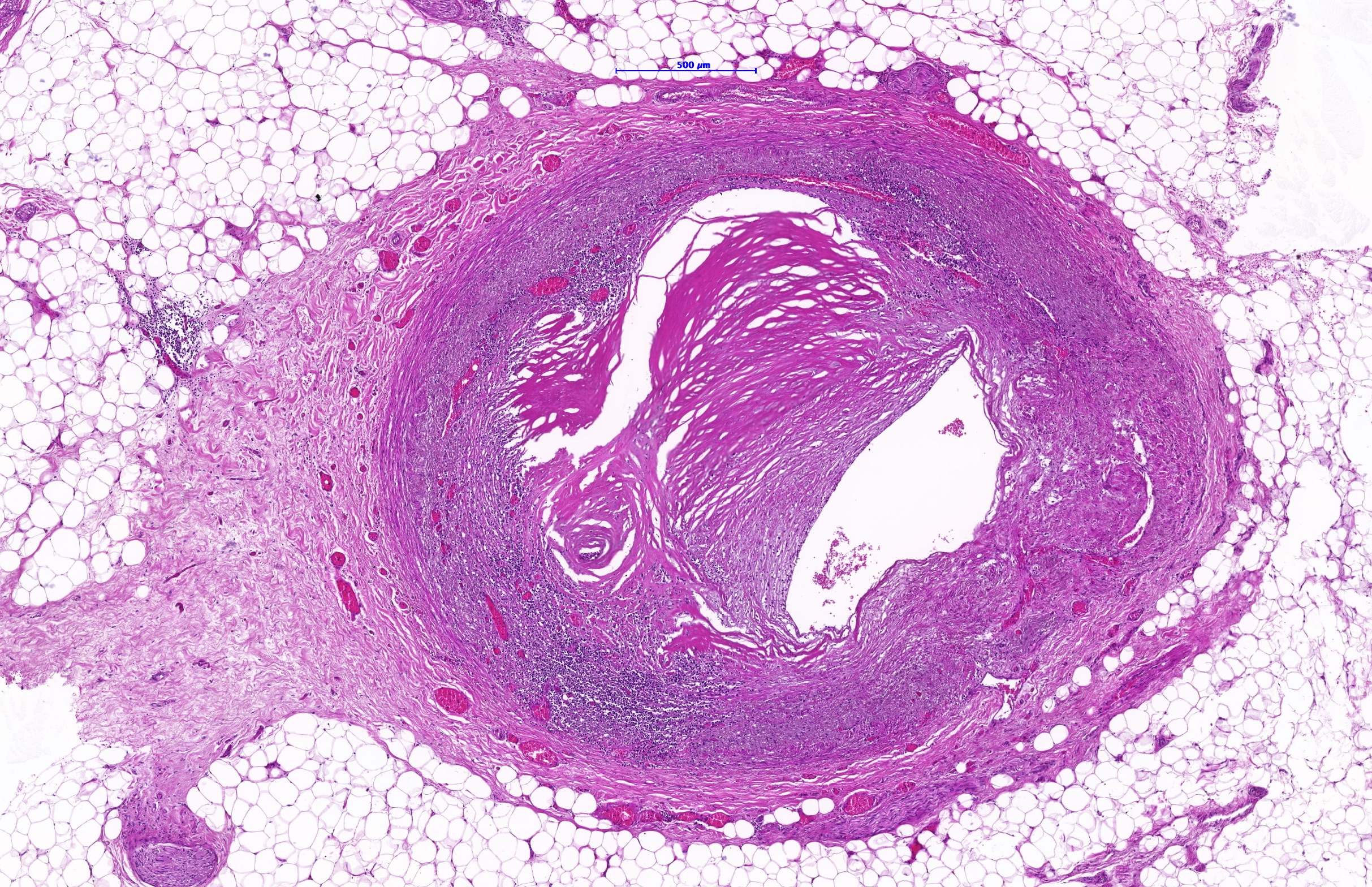

Supplement: Supplementary file 6 — Source data Fig. 5 [file 44319_2024_251_MOESM6_ESM.zip › Fig 5/Fig 5A/TAK 1802 copy.jpg]

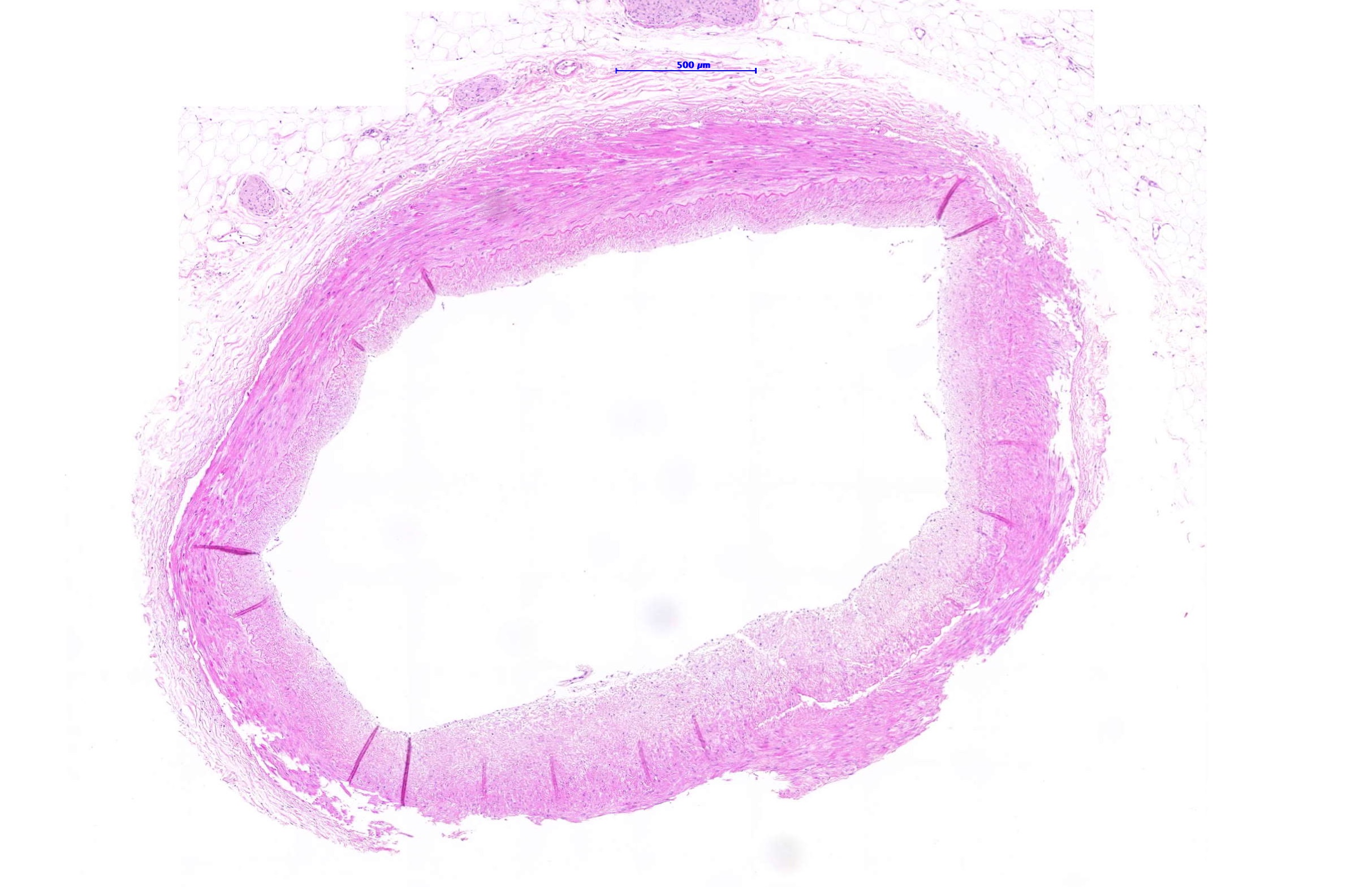

Supplement: Supplementary file 6 — Source data Fig. 5 [file 44319_2024_251_MOESM6_ESM.zip › Fig 5/Fig 5A/control 3.jpg]

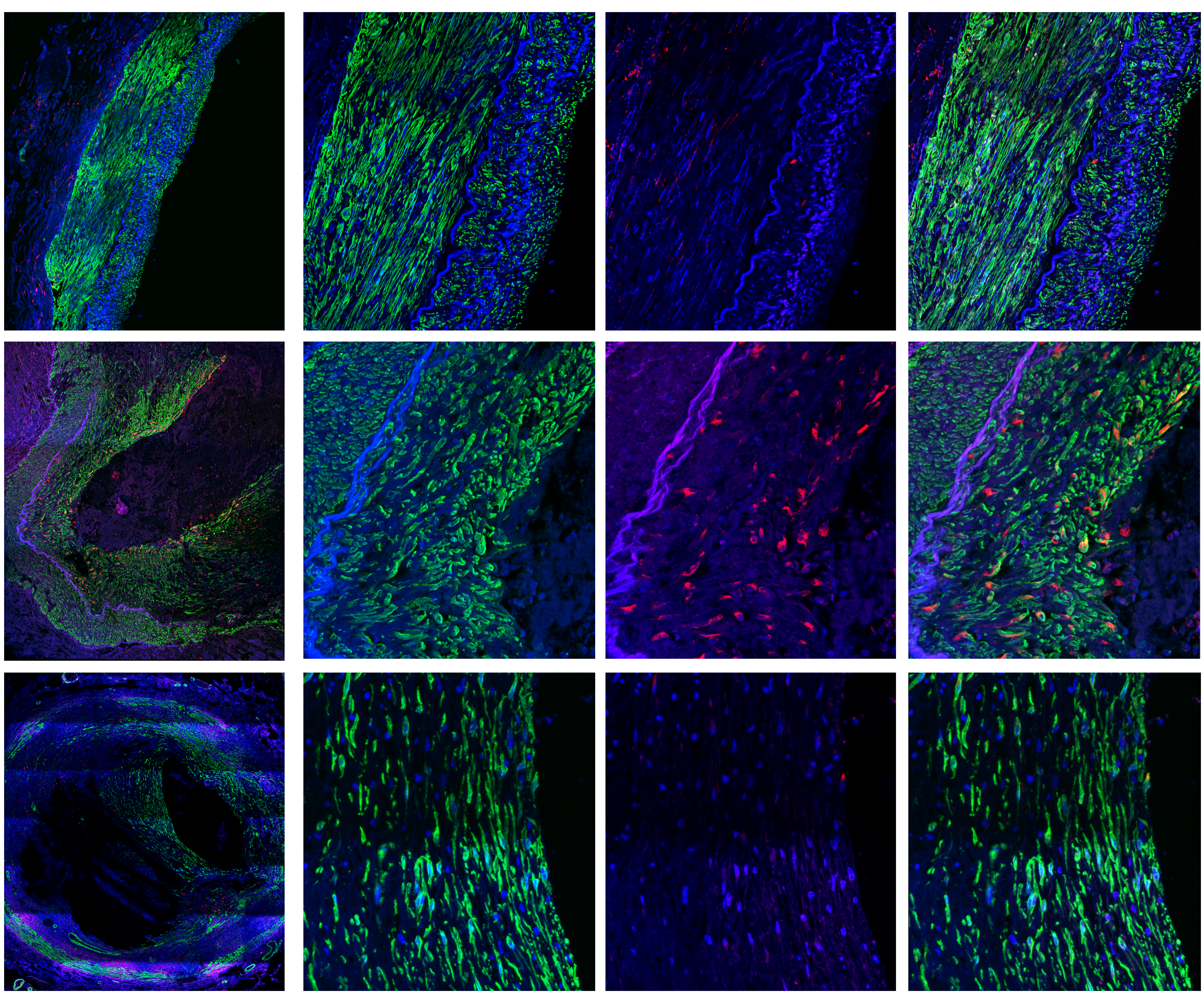

Supplement: Supplementary file 6 — Source data Fig. 5 [file 44319_2024_251_MOESM6_ESM.zip › Fig 5/Fig 5B/5B.tiff]

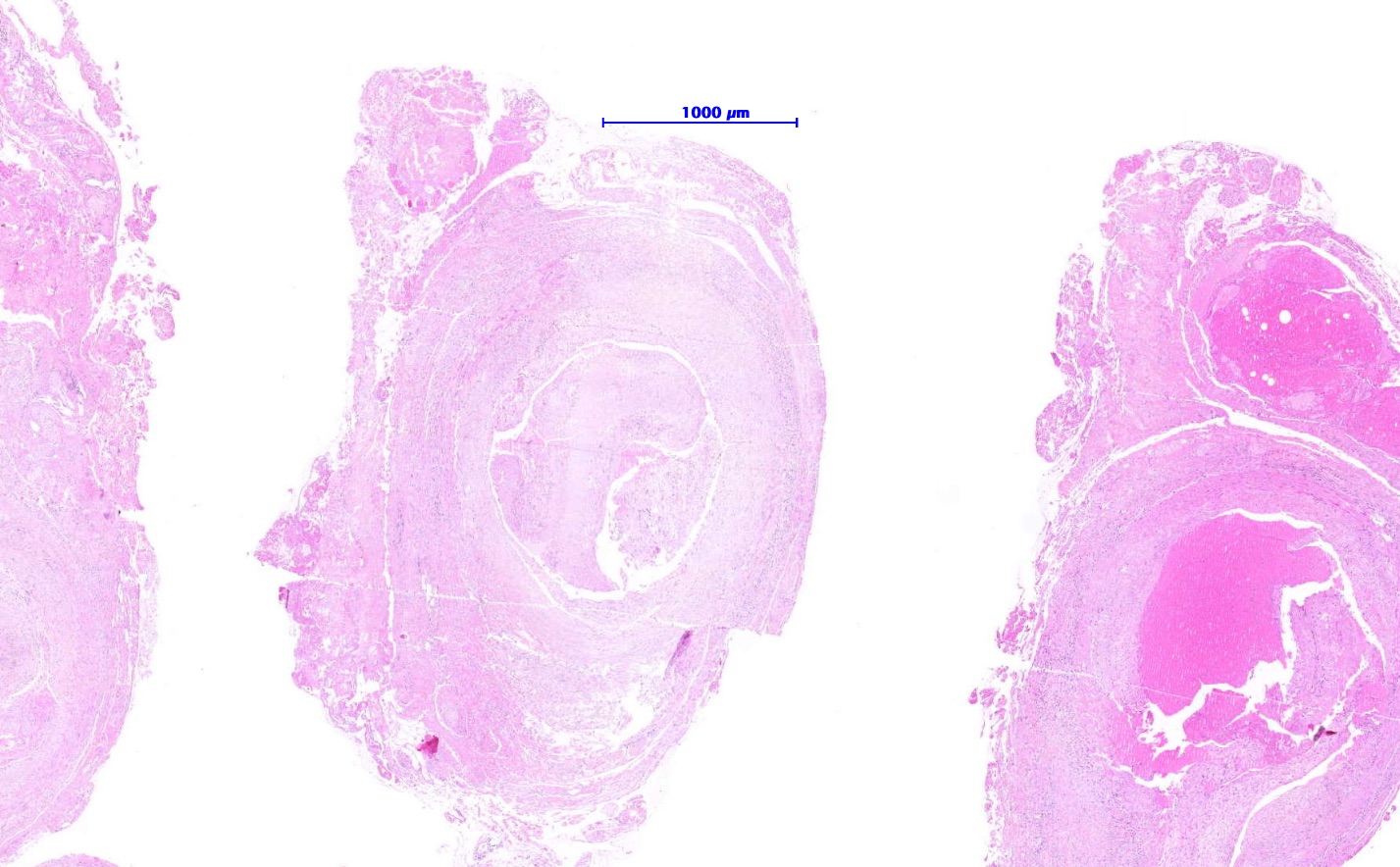

Supplement: Supplementary file 6 — Source data Fig. 5 [file 44319_2024_251_MOESM6_ESM.zip › Fig 5/Fig 5C/717 copy.jpg]

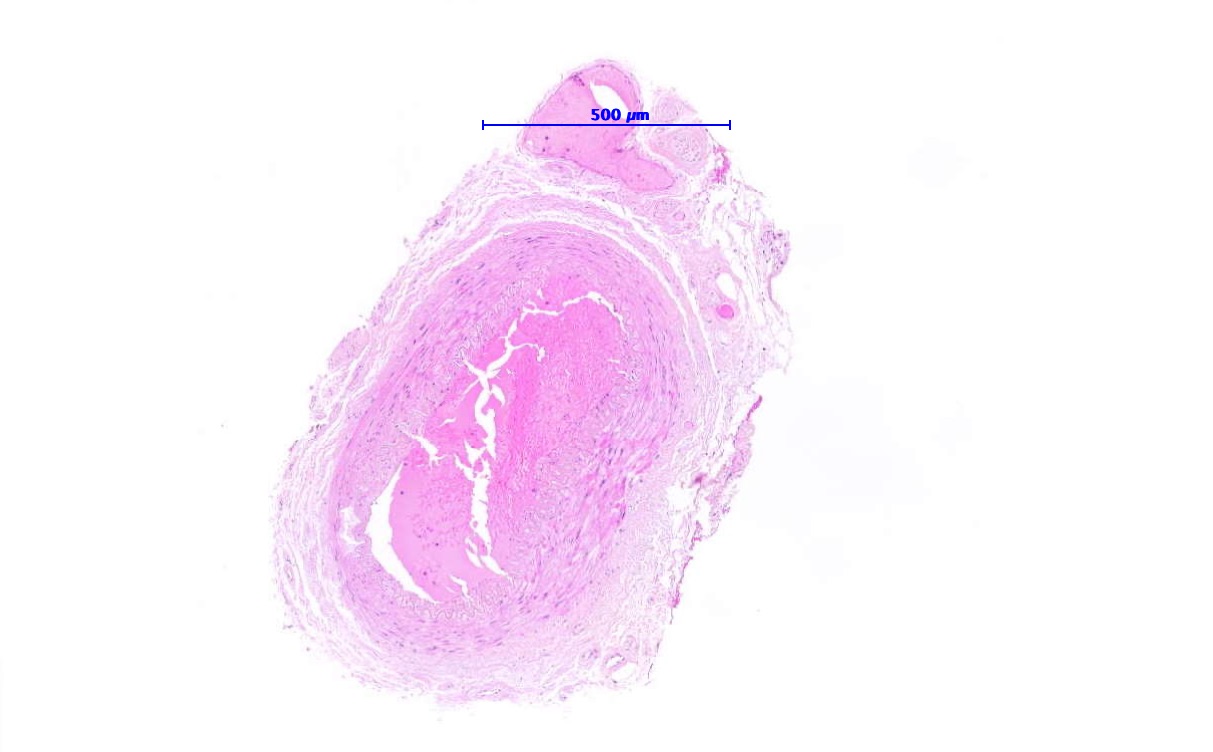

Supplement: Supplementary file 6 — Source data Fig. 5 [file 44319_2024_251_MOESM6_ESM.zip › Fig 5/Fig 5C/6574 normal copy.jpg]

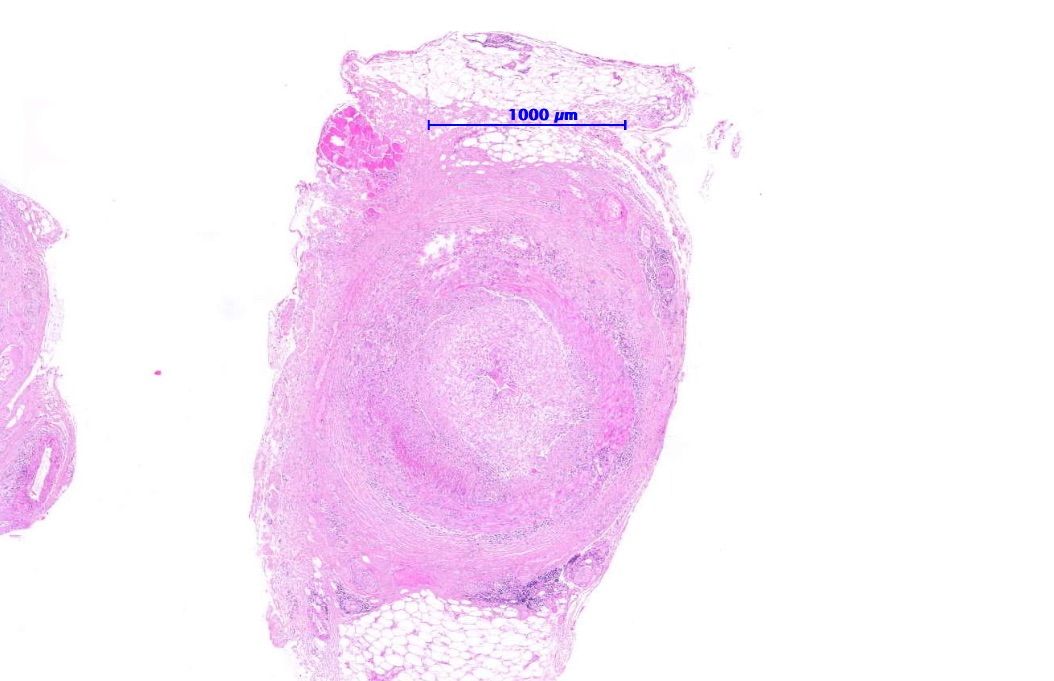

Supplement: Supplementary file 6 — Source data Fig. 5 [file 44319_2024_251_MOESM6_ESM.zip › Fig 5/Fig 5C/8054 copy.jpg]

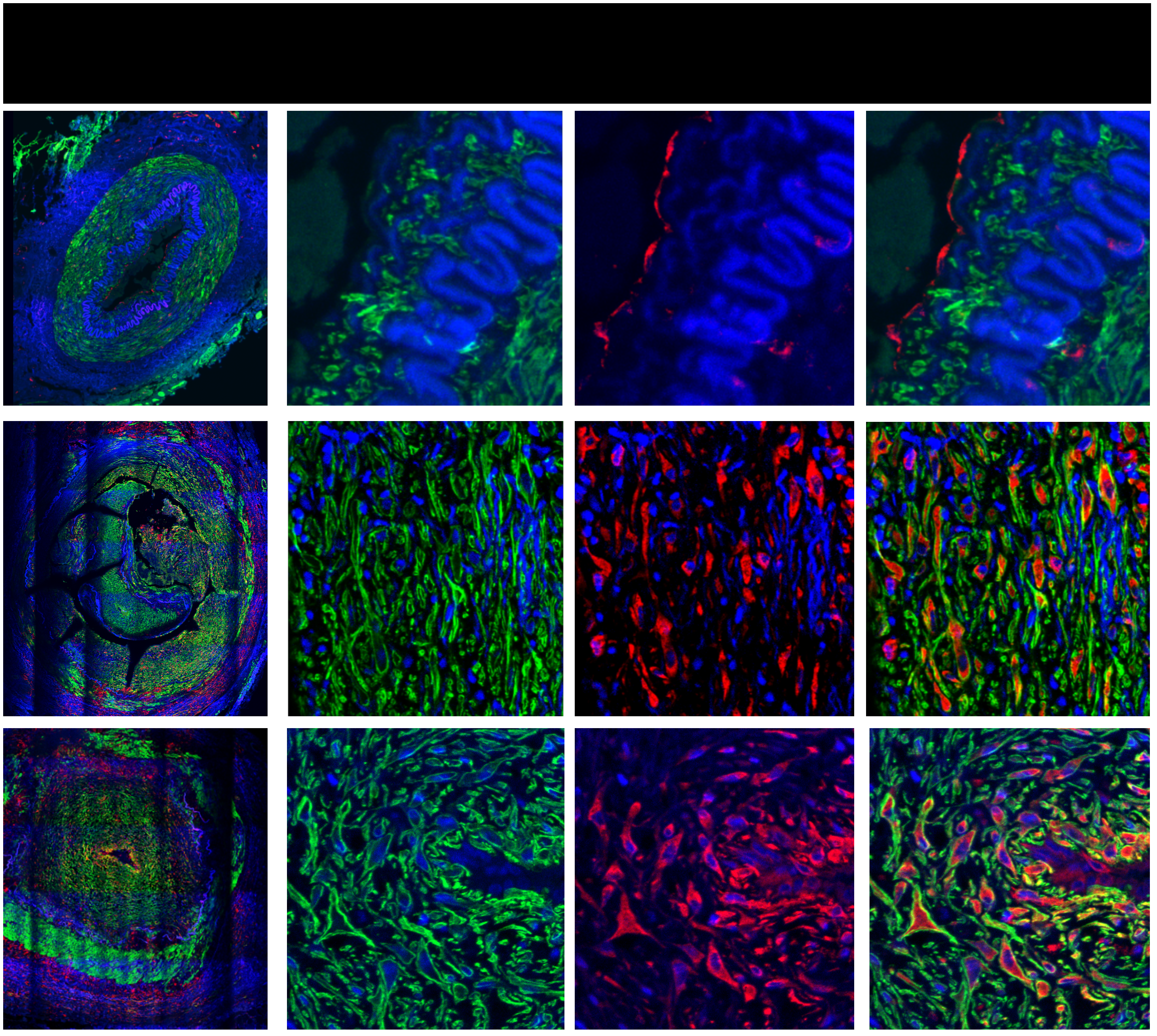

Supplement: Supplementary file 6 — Source data Fig. 5 [file 44319_2024_251_MOESM6_ESM.zip › Fig 5/Fig 5D/5D.tiff]

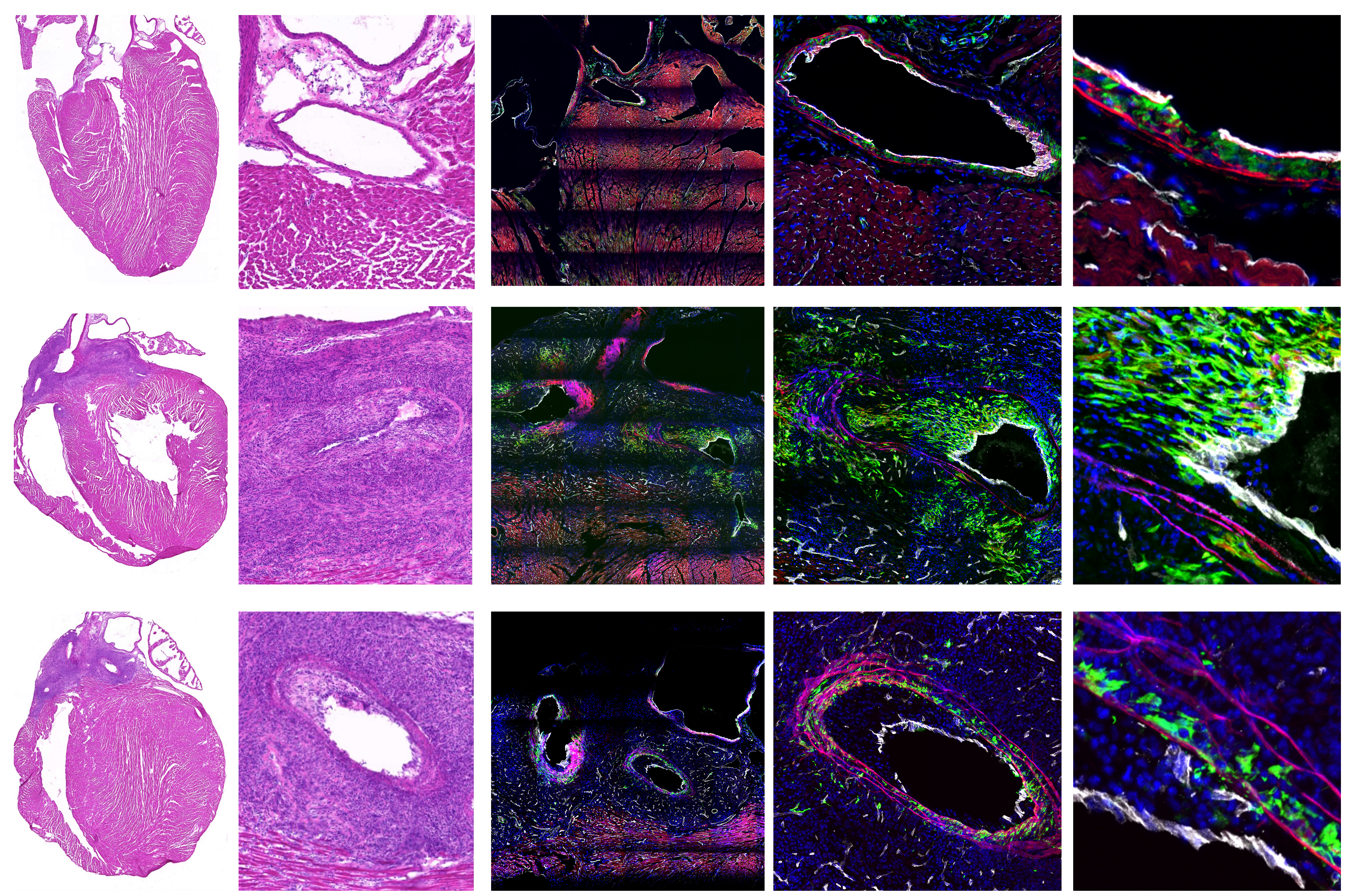

Supplement: Supplementary file 7 — Source data Fig. 6 [file 44319_2024_251_MOESM7_ESM.zip › Fig 6 Source data/Fig 6C:D/6C:D.tiff]

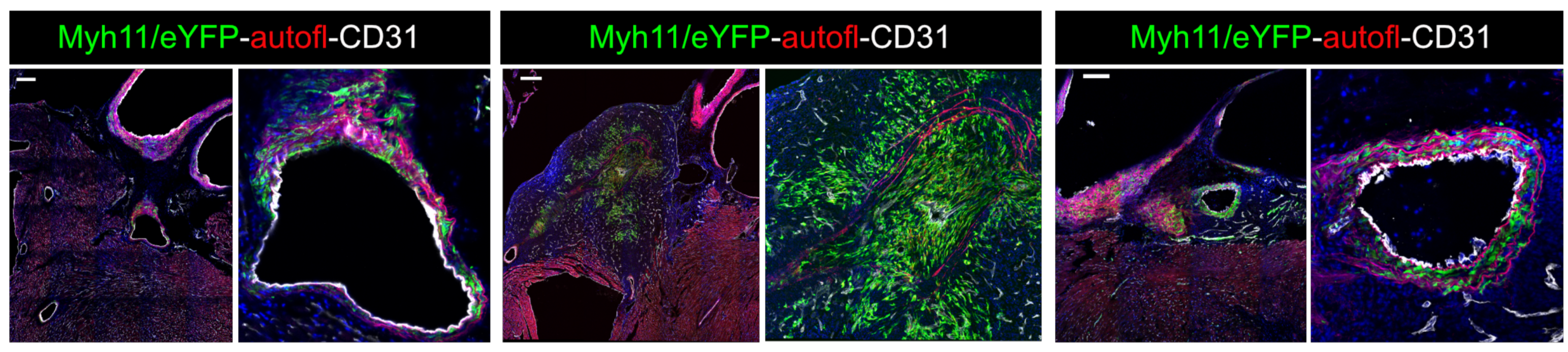

Supplement: Supplementary file 8 — Source data Fig. 7 [file 44319_2024_251_MOESM8_ESM.zip › Fig 7 B:C Source data/7B.tiff]

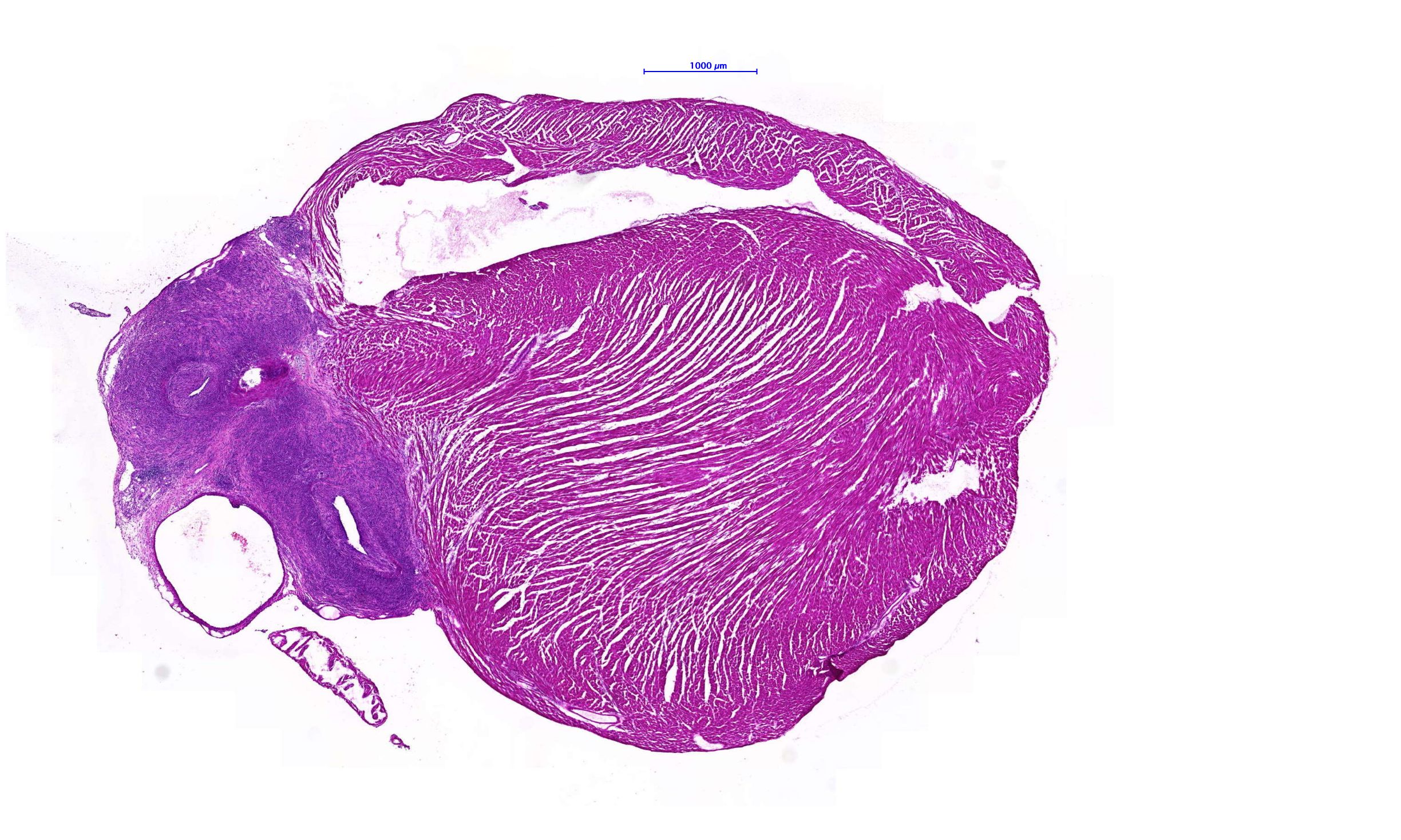

Supplement: Supplementary file 9 — Figure EV1 Source Data [file 44319_2024_251_MOESM9_ESM.zip › Fig EV1 Source data/Fig EV1A/CAWS CA whole copy.jpg]

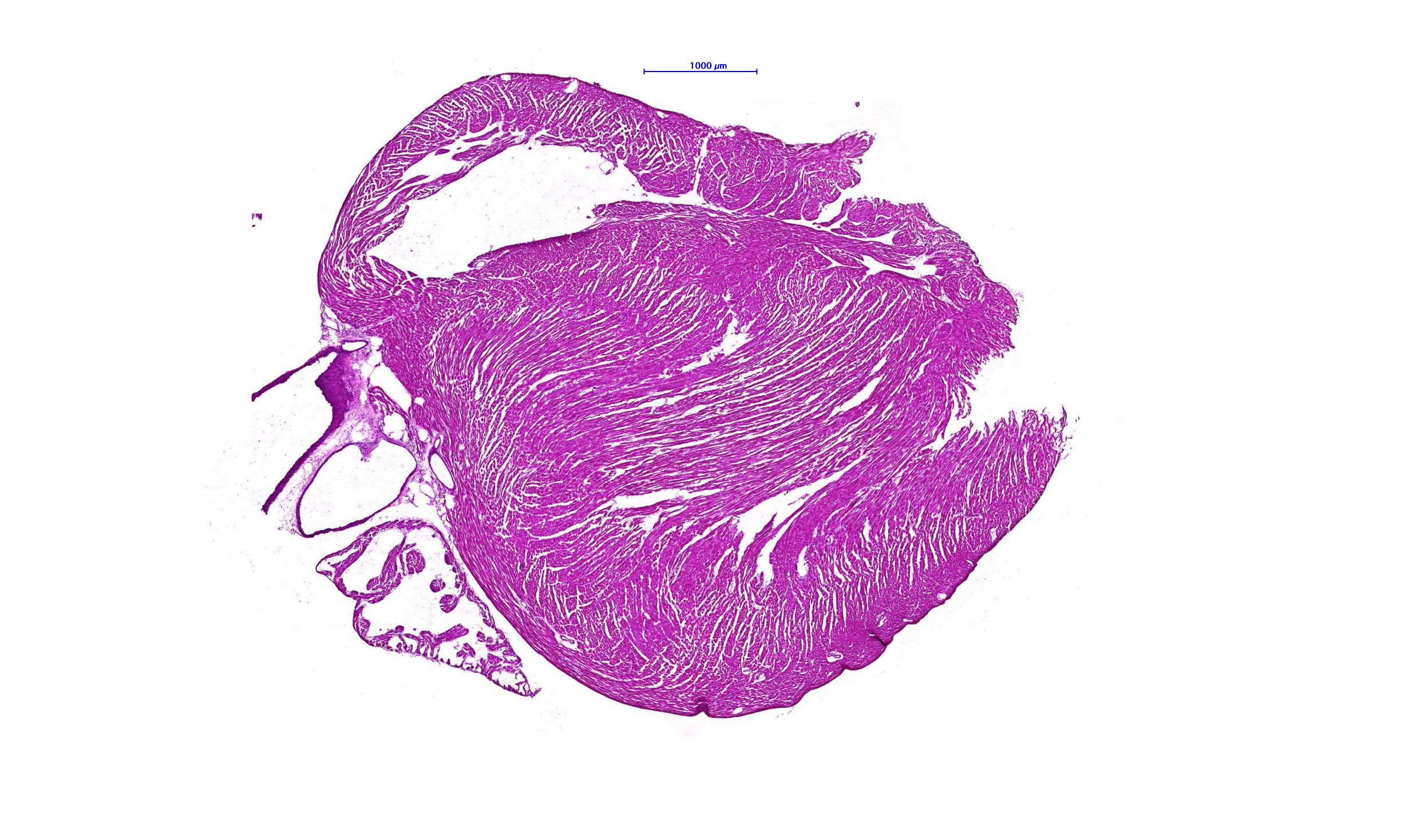

Supplement: Supplementary file 9 — Figure EV1 Source Data [file 44319_2024_251_MOESM9_ESM.zip › Fig EV1 Source data/Fig EV1A/Naive whole copy.jpg]

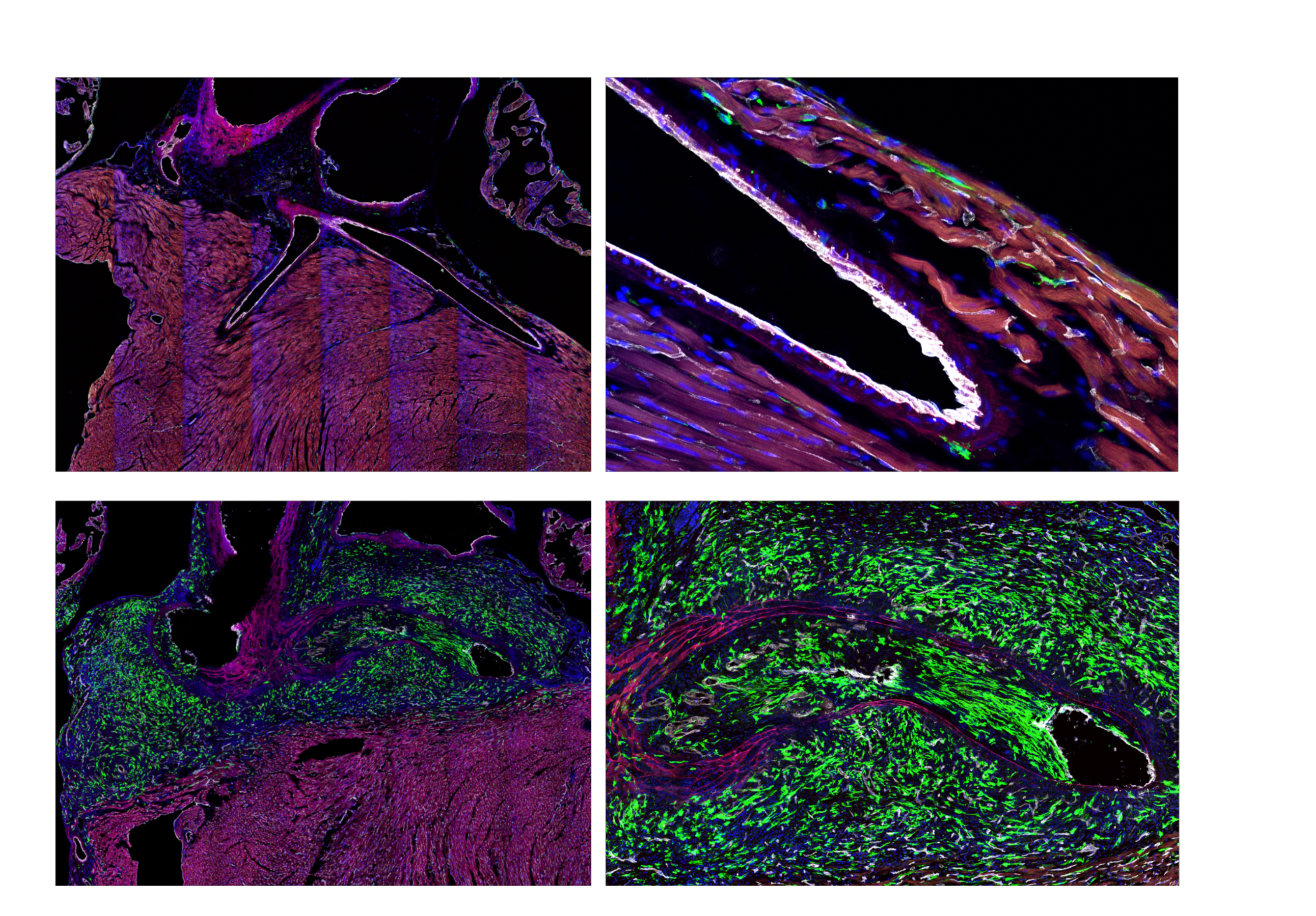

Supplement: Supplementary file 9 — Figure EV1 Source Data [file 44319_2024_251_MOESM9_ESM.zip › Fig EV1 Source data/Fig EV1F/Col1a2eYFP.tiff]
