# Supplementary material for: mTOR signalling controls the formation of smooth muscle cell-derived luminal myofibroblasts during vasculitis
Source: EMBO Rep. 2024 Sep 13;25(10):4570–93. doi: 10.1038/s44319-024-00251-1 (PMC11467406; doi:10.1038/s44319-024-00251-1)
Supplement: Supplementary file 11 — Expanded View Figures [file 44319_2024_251_MOESM11_ESM.pdf]

Expanded View Figures

Figure EV1. CAWS-induced vasculitis triggers the formation of collagen-expressing luminal myofibroblasts.

(**A**) Cardiac sections from naive or CAWS-injected mice (4–5 weeks post injection) analysed by histology or immunofluorescent microscopy. For the latter, sections were stained for CD31 to label endothelial cells and autofluorescence was used to identify elastin fibres of the media. (**B**) Graphs show the maximum width of the coronary artery intima for individual mice (with mean ± SEM) pooled from 3 independent experiments. (**C**) Genetic schema for $Col1a2^{CreERT2}.R26^{eYFP}$ system. (**D**) Flow cytometric analysis of cardiac cells from $Col1a2^{CreERT2}.R26^{eYFP}$ (and control) mice showing endogenous eYFP+ expression and PDGFRα staining. Inset value is the mean of 4–6 mice from 3 independent experiments. (**E**) Experimental schema. (**F**) Cardiac sections from naive or CAWS-injected $Col1a2^{CreERT2}.R26^{eYFP}$ mice stained for GFP to identify Col1a2 + /eYFP+ cells (green), CD31 to label endothelial cells (white) and autofluorescence to identify elastin fibres of the media (red). (**G**) Graphs show the number of Col1a2 + /eYFP+ cells within each vessel layer for individual mice (with mean ± SEM) pooled from 3 independent experiments. The coronary artery (CA), adventitia (A), media (M) and intima (I) are annotated and scale bars are 1000 µm. *** $P < 0.001$ with two-tailed Student's t tests. Exact P values (to 4 decimal points) for: (**B**) 0.0004 (***). Source data are available online for this figure.

 © The Author(s)

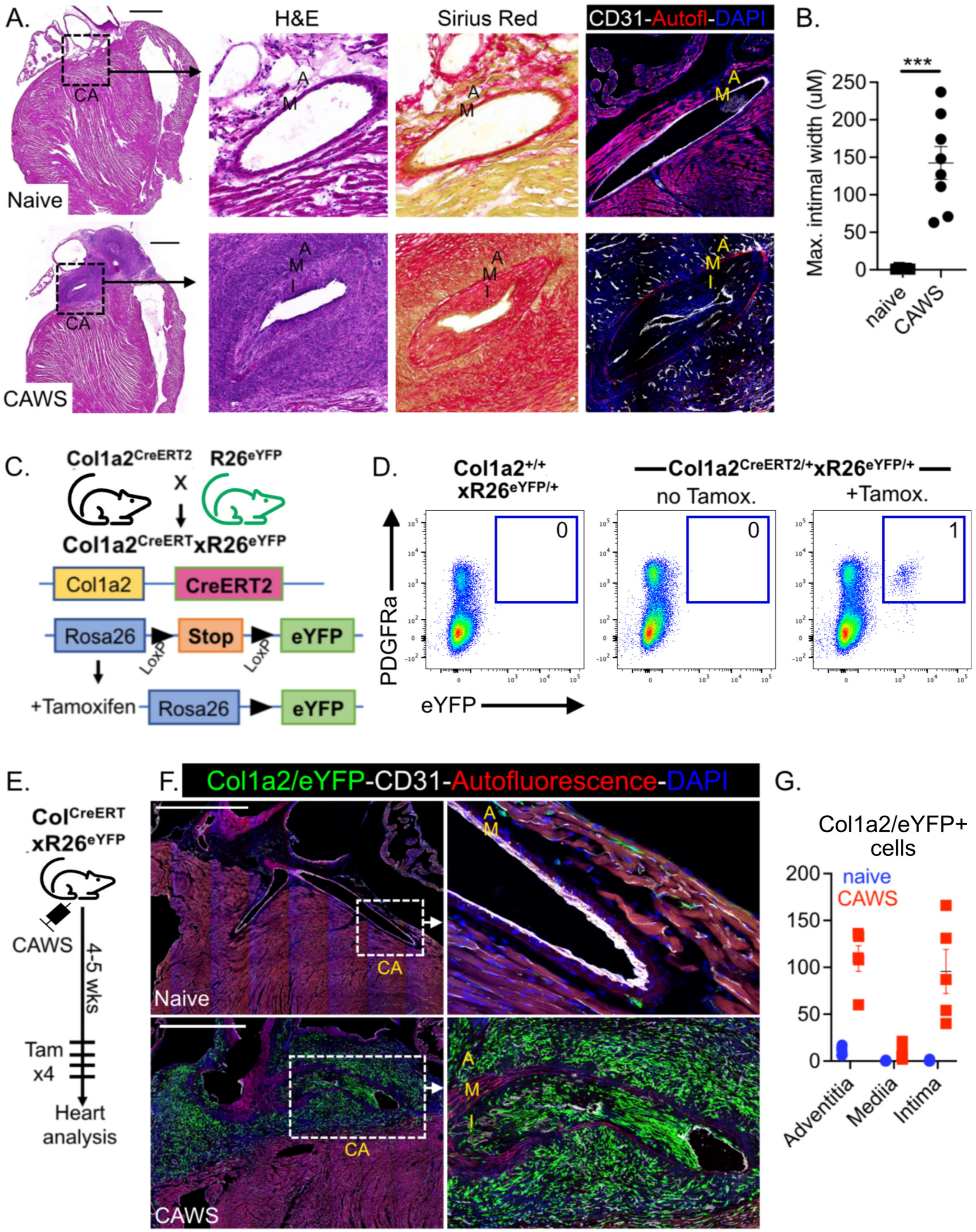

A.
H&E
Sirius Red
CD31-Autofl-DAPI
Naive
CAWS
CA
A
M
I
B.
***
Max. intimal width (uM)
250
200
150
100
50
0
naive
CAWS
C.
Col1a2CreERT2
R26eYFP
X
Col1a2CreERTxR26eYFP
Col1a2
CreERT2
Rosa26
Stop
eYFP
LoxP
+Tamoxifen
D.
Col1a2+/+ xR26eYFP/+
Col1a2CreERT2/+xR26eYFP/+
no Tamox.
+Tamox.
0
0
1
PDGFRa
eYFP
E.
ColCreERT xR26eYFP
CAWS
4-5 wks
Tam x4
Heart analysis
F.
Col1a2/eYFP-CD31-Autofluorescence-DAPI
G.
Col1a2/eYFP+ cells
naive
CAWS
Adventitia
Mediia
Intima

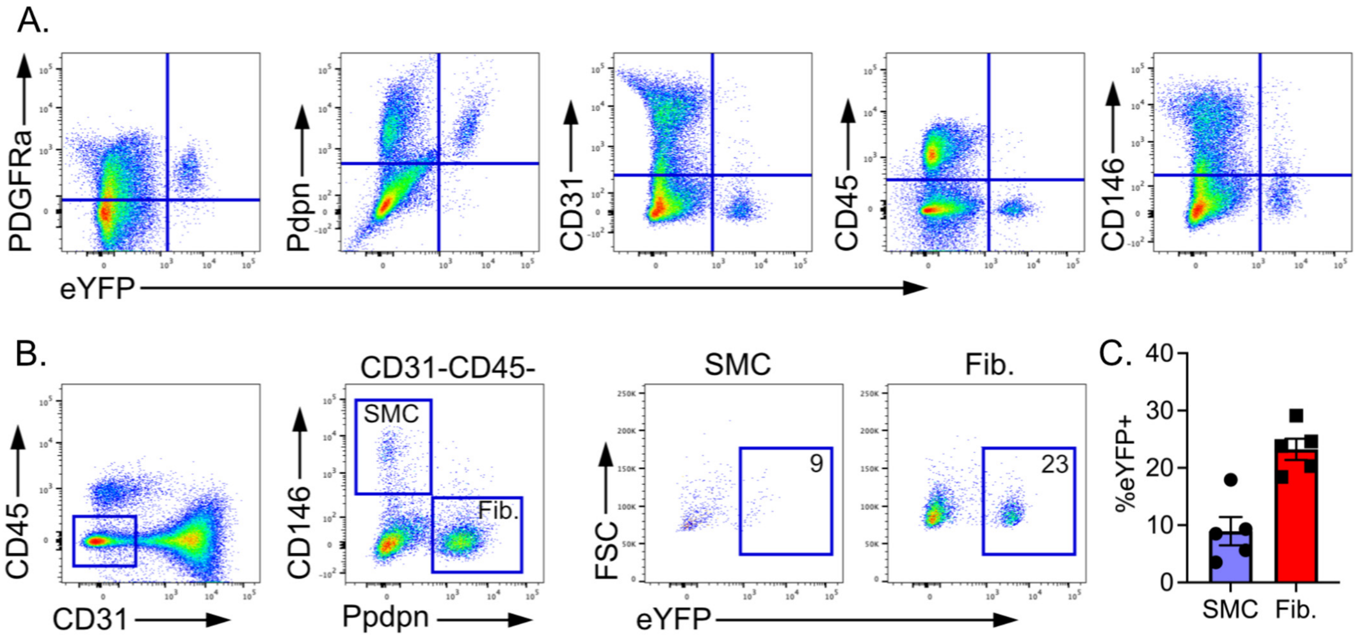

Figure EV2. E10.5 Wt1 recombination predominantly labels resident cardiac fibroblasts.

(**A**) Flow cytometric analysis of cardiac cells from $Wt1^{CreERT2}.R26^{eYFP}$ mice (administered tamoxifen at E10.5) showing endogenous eYFP+ expression versus lineage-specific markers. (**B**) Gating strategy and eYFP labelling of SMC (CD45-CD31-CD146 +) and fibroblasts (CD45-CD31-Pdpn +). (**C**) Graphs show the proportion of eYFP labelling in SMCs and fibroblasts for individual mice (with mean ± SEM) pooled from 2 independent experiments. Source data are available online for this figure.

 © The Author(s)

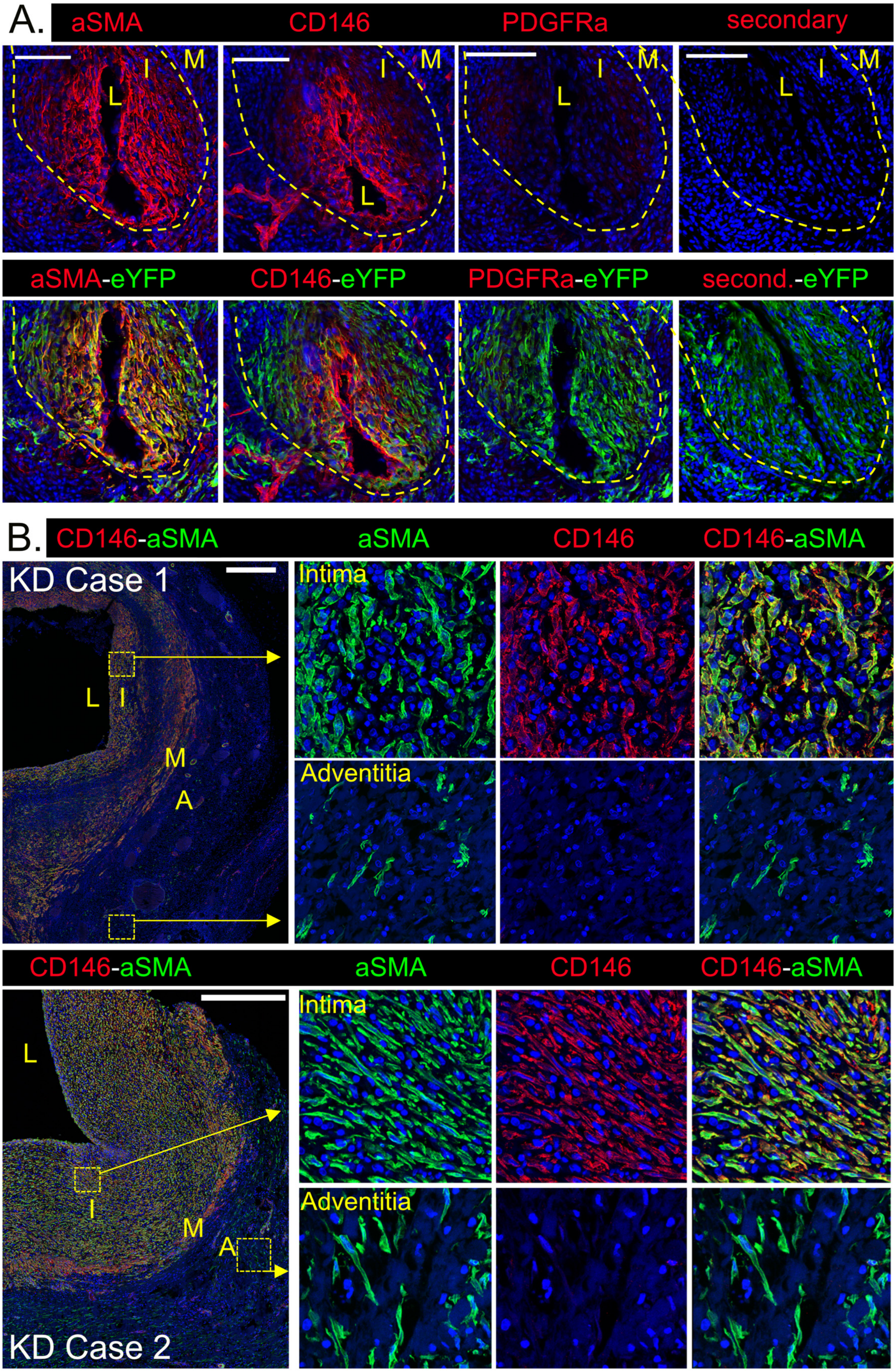
A.
aSMA
CD146
PDGFRa
secondary
I
M
L
aSMA-eYFP
CD146-eYFP
PDGFRa-eYFP
second.-eYFP
B.
CD146-aSMA
aSMA
CD146
CD146-aSMA
KD Case 1
Intima
Adventitia
L
I
M
A
KD Case 2

Figure EV3. Luminal myofibroblasts express mural cell markers.

(**A**) Cardiac sections from CAWS-injected $Myh11^{CreERT2}.R26^{eYFP}$ mice (4–5 weeks post CAWS) analysed by immunofluorescent microscopy. Sections were stained for GFP to identify Myh11+/eYFP+ cells (green) and either α-SMA, CD146 or PDGFRα (red). Scale bars are 100 μm. (**B**) Coronary artery sections from two acute KD fatalities stained for α-SMA (green) and CD146 (red) and analysed by confocal microscopy. Scale bars are 1000 μm. The adventitia (A), media (M), intima (I) and lumen (L) are annotated throughout. Source data are available online for this figure.

 © The Author(s)

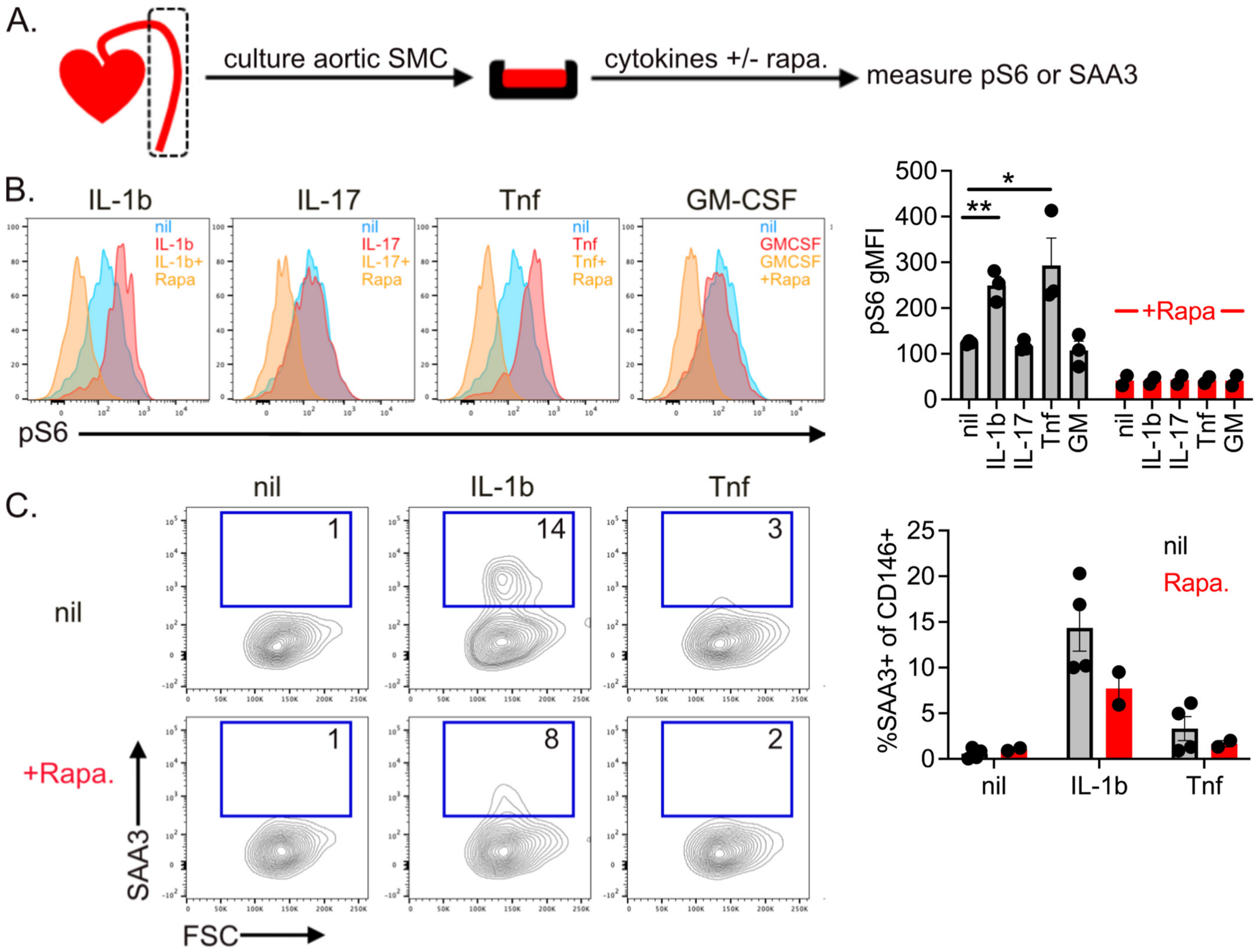

Figure EV4. IL-1β activates mTOR in SMCs in vitro.

(**A**) Experimental schematic. (**B**, **C**) FACS plots are gated on CD146+ cells and show intracellular staining for pS6 (**B**) or SAA3 (**C**) in cultured SMCs stimulated with cytokines +/− rapamycin. Graphed points depict individual lines (with mean ± SEM) pooled from 2 independent experiments. *$P < 0.05$; **$P < 0.01$ with two-tailed Student's *t* tests. Exact *P* values (to 4 decimal points) for (**B**) IL-1β 0.0032 (**), Tnf 0.0482 (*). Source data are available online for this figure.

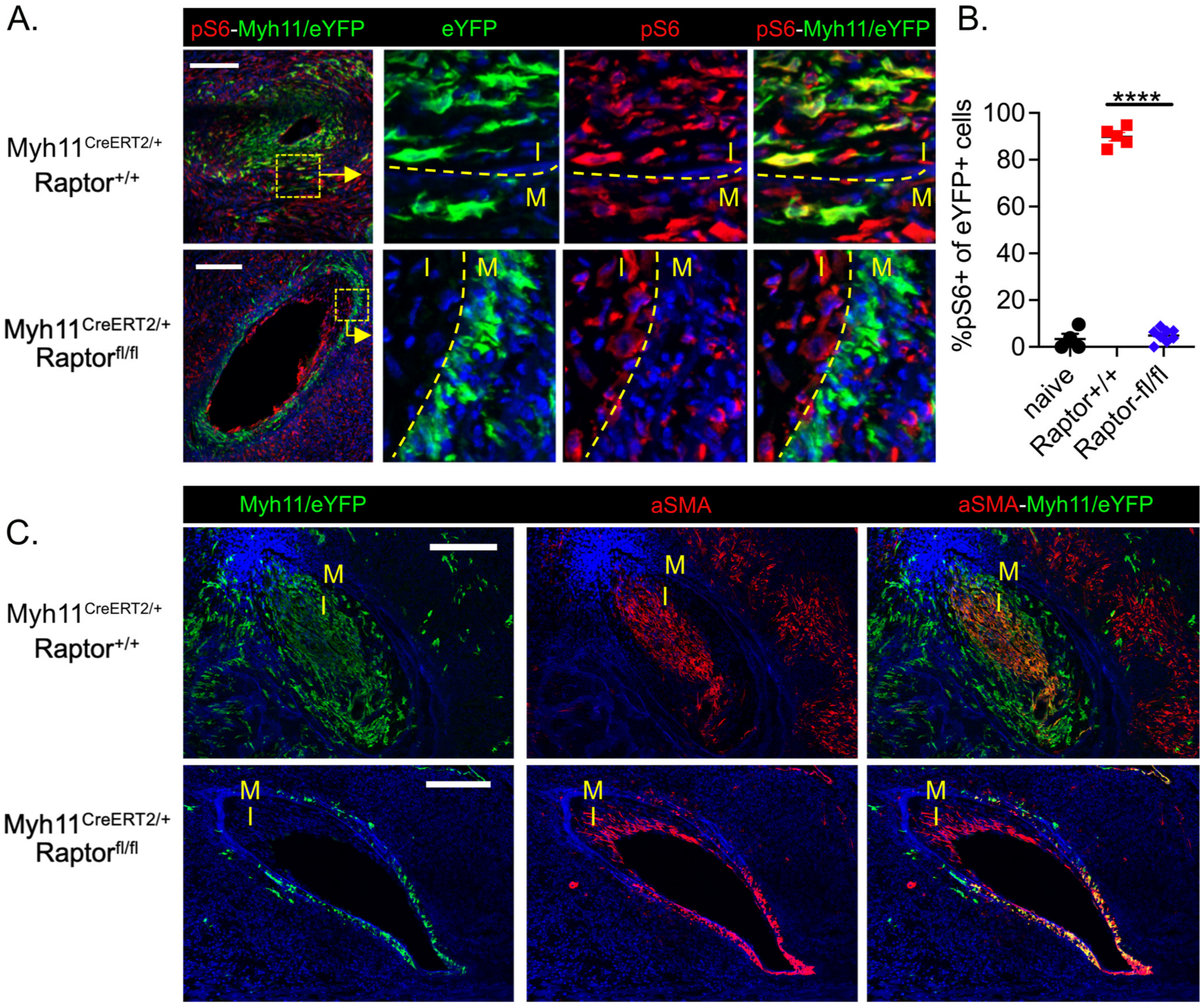

Figure EV5. Analysis of Raptor-deficient SMCs.

(**A–C**) Representative cardiac sections from CAWS-injected $Myh11^{CreERT2}$.$Raptor^{+/+}$.$R26^{eYFP}$ or $Myh11^{CreERT2}$.$Raptor^{fl/fl}$.$R26^{eYFP}$ mice (4–5 weeks post injection). (**A**, **B**) Sections are stained for GFP to identify Myh11+/eYFP+ cells (green) and pS6 (red) to assess mTOR signalling. Graphs depict the % eYFP+ cells that are pS6+ for individual mice (with mean ± SEM) pooled from 3 independent experiments. (**C**) Sections show eYFP+ cells and α-SMA expression. The IEL is shown as a dashed line in (**A**) and the adventitia (A), media (M), intima (I) and lumen (L) are annotated throughout. Scale bars are 100 μm. ****$P < 0.0001$ with two-tailed Student's *t* tests. Exact *P* values (to 4 decimal points) for (**B**) < 0.0001 (****). Source data are available online for this figure.

 © The Author(s)
